# OP69 Are Artificial-Intelligence-Based Literature Reviews Accepted By Health Technology Assessment Bodies?

**DOI:** 10.1017/S0266462324001284

**Published:** 2025-01-07

**Authors:** Gautamjeet Singh-Mangat, Sugandh Sharma, Rito Bergemann

## Abstract

**Introduction:**

Literature reviews (LR) play a crucial role in all health technology assessment (HTA) dossiers, presenting evidence-based value of interventions. There is global exploration of artificial intelligence (AI) to expedite and enhance the efficiency of literature reviews. Our research aimed to identify any existing guidance from HTA bodies regarding the use of AI for conducting literature reviews.

**Methods:**

We conducted a comprehensive search and review of any published guidance from prominent HTA bodies, including the National Institute for Health and Care Excellence (NICE, England), Scottish Medicines Consortium (SMC, Scotland), National Centre for Pharmacoeconomics (NCPE, Ireland), National Authority for Health (HAS, France), Federal Joint Committee (G-BA, Germany), Institute for Quality and Efficiency in Health Care (IQWiG, Germany), Canadian Agency for Drugs and Technologies in Health (CADTH, Canada), and Pharmaceutical Benefits Advisory Committee (PBAC, Australia). This was done to gain insights into their views regarding the utilization of AI in literature reviews. Additionally, we engaged with HTA representatives, such as NICE, to gain a deeper understanding of their perspectives.

**Results:**

We found a lack of clear guidance on the use of AI for conducting LRs. NICE has recommended a priority screening technique using machine learning (ML) for identification of a higher proportion of relevant papers at an earlier stage. NICE is currently in the process of developing guidance and is updating its manual in this area. SMC refers readers to NICE methodologies. In its HRB-CICER report, NCPE only acknowledges the potential of ML algorithms for LRs, with no additional information. IQWiG, in its general methods, recommends the use of ML-validated classifiers for identifying randomized controlled trials (RCTs) within bibliographic searches.

**Conclusions:**

Our research indicates that there is scarce guidance available for the use of AI in LRs for HTA submissions. However, considering the rapidly evolving nature of this field, it is anticipated that guidance documents and manuals will be updated in the near future.

